# Influence of Brain Stem on Axial and Hindlimb Spinal Locomotor Rhythm Generating Circuits of the Neonatal Mouse

**DOI:** 10.3389/fnins.2018.00053

**Published:** 2018-02-09

**Authors:** Céline Jean-Xavier, Marie-Claude Perreault

**Affiliations:** Department of Physiology, Emory University School of Medicine, Atlanta, GA, United States

**Keywords:** motor command, subcortical, reticulospinal, vestibulospinal, CPG, trunk-hindlimb coordination

## Abstract

The trunk plays a pivotal role in limbed locomotion. Yet, little is known about how the brain stem controls trunk activity during walking. In this study, we assessed the spatiotemporal activity patterns of axial and hindlimb motoneurons (MNs) during drug-induced fictive locomotor-like activity (LLA) in an isolated brain stem-spinal cord preparation of the neonatal mouse. We also evaluated the extent to which these activity patterns are affected by removal of brain stem. Recordings were made in the segments T7, L2, and L5 using calcium imaging from individual axial MNs in the medial motor column (MMC) and hindlimb MNs in lateral motor column (LMC). The MN activities were analyzed during both the rhythmic and the tonic components of LLA, the tonic component being used as a readout of generalized increase in excitability in spinal locomotor networks. The most salient effect of brain stem removal was an increase in locomotor rhythm frequency and a concomitant reduction in burst durations in both MMC and LMC MNs. The lack of effect on the tonic component of LLA indicated specificity of action during the rhythmic component. Cooling-induced silencing of the brain stem reproduced the increase in rhythm frequency and accompanying decrease in burst durations in L2 MMC and LMC, suggesting a dependency on brain stem neuron activity. The work supports the idea that the brain stem locomotor circuits are operational already at birth and further suggests an important role in modulating trunk activity. The brain stem may influence the axial and hindlimb spinal locomotor rhythm generating circuits by extending their range of operation. This may represent a critical step of locomotor development when learning how to walk in different conditions and environments is a major endeavor.

## Introduction

Locomotion in mammals is efficient when both trunk and hindlimbs are appropriately controlled (Carlson et al., 1979; Thorstensson et al., 1982; Gramsbergen, 1998; Jamon, 2006; Schilling, 2011). Evidence indicates that both the brainstem and the spinal cord participate in the control of trunk and hindlimb activity during locomotion (Musienko et al., 2014). However, little is known about the respective contribution of these two control systems.

Our understanding of the circuits controlling locomotor activity in trunk muscles relies heavily on studies in vertebrates that either have no legs or only simple forms of hindlimbs, including lamprey (Grillner, 2006; Ryczko et al., 2010), xenopus (Combes et al., 2004; Beyeler et al., 2008; Roberts et al., 2012), leech (Kristan et al., 2005), zebrafish (Thorsen, 2004; Bagnall and McLean, 2014; Kishore et al., 2014; Grillner and Manira, 2015), and amphibian tetrapods (Cabelguen et al., 2014; Ryczko et al., 2015). Recent work in rodents suggests that some of the basic mechanisms that underlie control of trunk in limbless vertebrates are retained in limbed mammals (Falgairolle et al., 2013; Beliez et al., 2015; Hinckley et al., 2015). However, the more complex mechanisms that have developed along with the specialization that came with the morphological evolution of the hindlimbs (Beyeler et al., 2008; Le Gal et al., 2016) have yet to be studied.

The present study in a brain stem-spinal cord preparation of the neonatal mouse was designed to examine the contribution of the brain stem to trunk and hindlimb motoneurons (MNs) activities during drug-induced fictive locomotor-like activity (LLA; Kudo and Yamada, 1987; Smith and Feldman, 1987; Jiang et al., 1999; Bonnot et al., 2002). We used calcium imaging to record locomotor activity from individual MNs in T7, L2, or L5 segments and compare recordings from MNs in the medial and lateral motor column (MMC and LMC) before and after removal of the brain stem. This approach enables before/after comparison in the same preparation, making for a more robust interpretation of the results.

The findings suggest an early influence of the brain stem directed to the axial and hindlimb spinal locomotor rhythm generating circuits. This early influence may be critical when animals learn how to walk in different conditions and environment. Preliminary results have been published previously in abstract form (Jean-Xavier and Perreault, 2013).

## Materials and methods

### Animals

Experiments were performed on neonatal [post-natal day (P) 0–2] ICR/Ha mice (*n* = 36). All animal protocols followed US National Institutes of Health guidelines and were approved by Emory University Institutional Animal Care and Use Committee.

### *In vitro* brain stem-spinal cord preparation

Under deep isoflurane (4%) anesthesia, animals were decerebrated by transecting the brain rostral to the superior colliculus and eviscerated. Preparations were then placed in a dissection chamber filled with ice cold oxygenated (95% O_2_/5% CO_2_) glycerol-based dissecting solution containing (in mM): glycerol 250, KCl 2, D-glucose 11, CaCl_2_ 0.15, MgSO_4_ 2, NaH_2_PO_4_ 1.2, HEPES 5 and NaHCO_3_ 25 (pH of 7.4). After a craniotomy and a laminectomy, the brain stem-spinal cords, with the dorsal and ventral roots attached, were gently dissected out. Brain stem-spinal cords were then transferred to a Sylgard-coated recording chamber where they were positioned ventral side up. The recording chamber was partitioned into a brain stem and a spinal cord compartments using a plastic wall sealed with petroleum jelly at cervical segment C1-C2. Room temperature (RT; 23°C) oxygenated artificial cerebrospinal fluid (aCSF) containing (in mM): NaCl 128, KCl 3, D-glucose 11, CaCl_2_ 2.5, MgSO_4_ 1, NaH_2_PO_4_ 1.2, HEPES 5 and NaHCO_3_ 25, was pumped into each compartment. The tightness of the seal was verified by adding phenol red to one of the compartment.

### Loading of motoneurons with fluorescent calcium indicator

Spinal segments were identified by counting the ventral roots using as a starting point the C1 ventral root and/or the L5 ventral root which is last large diameter root of the lumbar enlargement. Motoneurons (MNs) in the thoracic segment T7 and lumbar segments L2 and L5 were loaded with Calcium Green 1-conjugated dextran amine (CaGDA; 3000 MW; Molecular Probes, for indicator kinetics (see Zhao et al., 1996; Kreitzer et al., 2000; Putney, 2006) by applying reconstituted CaGDA crystals (Glover, 1995) to the cut end of the corresponding ventral roots. Labeling via retrograde axonal transport continued in the dark at RT for at least 3 h.

### Drug-induced fictive locomotor-like activity (LLA)

LLA was induced by applying a neurochemical cocktail composed of N-Methyl-D-aspartate (5 μM NMDA), 5-hydroxytryptamine hydrochloride (10 μM 5-HT) and dopamine hydrochloride (50 μM DA) dissolved in aCSF. The locomotor cocktail was applied specifically to the spinal cord compartment (recycling flow rate of 15 ml/min). All neurochemicals were purchased from Sigma Aldrich and kept as frozen (−30°C) stock (10–100 mM).

### Removal of the brain stem

The brain stem was removed by complete transection of the spinal cord at the level of C1 segment using superfine Vannas scissors (WPI, USA). To avoid transecting during states of high excitability, neurochemicals were washed out for ≥20 min prior to transection. The locomotor cocktail solution was re-applied to the isolated spinal cord only after a post-transection recovery period of ≥20 min.

### Reversible cooling of the brain stem

In a subset of experiments, the excitability of the brain stem was reduced using cooling. For these experiments, temperature probes (Yellow Spring Instruments, YSI-402) were placed in each bath compartment. Prior to cooling, the brain stem compartment was set to an initial temperature of 28°C using warm aCSF. The brain stem was then cooled down to 18°C using ice-cold aCSF. After cooling, but before final removal of the brain stem, the brain stem compartment was rewarmed to 28°C. The spinal cord compartment was kept at RT at all times. Isothermal values were reached in 5 min.

### Calcium imaging

Drug-induced LLA is usually monitored using electrical recording from ventral roots which contain mixed axonal populations (axons from axial and limb MNs and sympathetic preganglionic neurons). Here, we used calcium imaging, which enable us to resolve activity in individual MNs identified as axial MNs of the medial motor column (MMC) or limb MNs of the lateral motor column (LMC) based on their mediolateral locations (Lev-Tov and O'Donovan, 1995; O'Donovan et al., 2008; Szokol and Perreault, 2009; Hinckley et al., 2015). Individual CaGDA-labeled MNs were visualized through the ventral white matter up to about 100 μm under the surface (Szokol and Perreault, 2009) using a 40x water immersion objective (LUMPLFLN 40X, 0.8 NA, Olympus USA) of an epi-fluorescence microscope (BX51, Olympus, USA) equipped with a 100 W halogen lamp driven by a DC power supply (PAN35-20A, Kikusui Electronics Corporation, Japan) and excitation and emission filters (BP 450–490 nm and LP 515 nm, respectively). Fluorescence images were captured using a sCMOS camera (PCO.edge, PCO, Canada) mounted on a video zoom adapter set at 0.5x. Image (16 bit) streams (480 frames) were stored at 4 frames/s (binning 2 × 2, gain 1) using the acquisition software Metamorph (v7.7, Universal Imaging Corporation, Molecular Devices, USA). All recording sessions (controls and trials) lasted 120 s.

### Data analysis

Using the acquisition and image analysis software Metamorph, circular regions of interest (ROIs) were positioned over the soma of individual MNs so as to cover as much of the field of view as possible. The selection of the MN somata was made according to labeling intensity and availability in the same focus plane. Fluorescence intensity within each ROI averaged over all pixels. These data were converted to text files and exported to pClamp (Clampfit 10.4, Molecular Devices, USA) where they were expressed as waveforms or changes in fluorescence over time for further analysis. Changes in fluorescence were measured and reported as percent changes from an average baseline level F0 [ΔF/F or (F–F0)/F0]. For the recordings that contained the onset of LLA (see “Tonic component” section below), F0 was measured prior to the arrival of the neurochemicals in the bath and thus estimated well the true baseline calcium level (“rest” period in absence of neurochemicals). For the recordings that contained only rhythmic activity (see “Rhythmic component” section below), F0 was measured as the minimum inter-burst fluorescence and thus likely overestimated the true baseline calcium level.

#### Locomotor-like activity (LLA)

##### Tonic component

**Figure 1:**
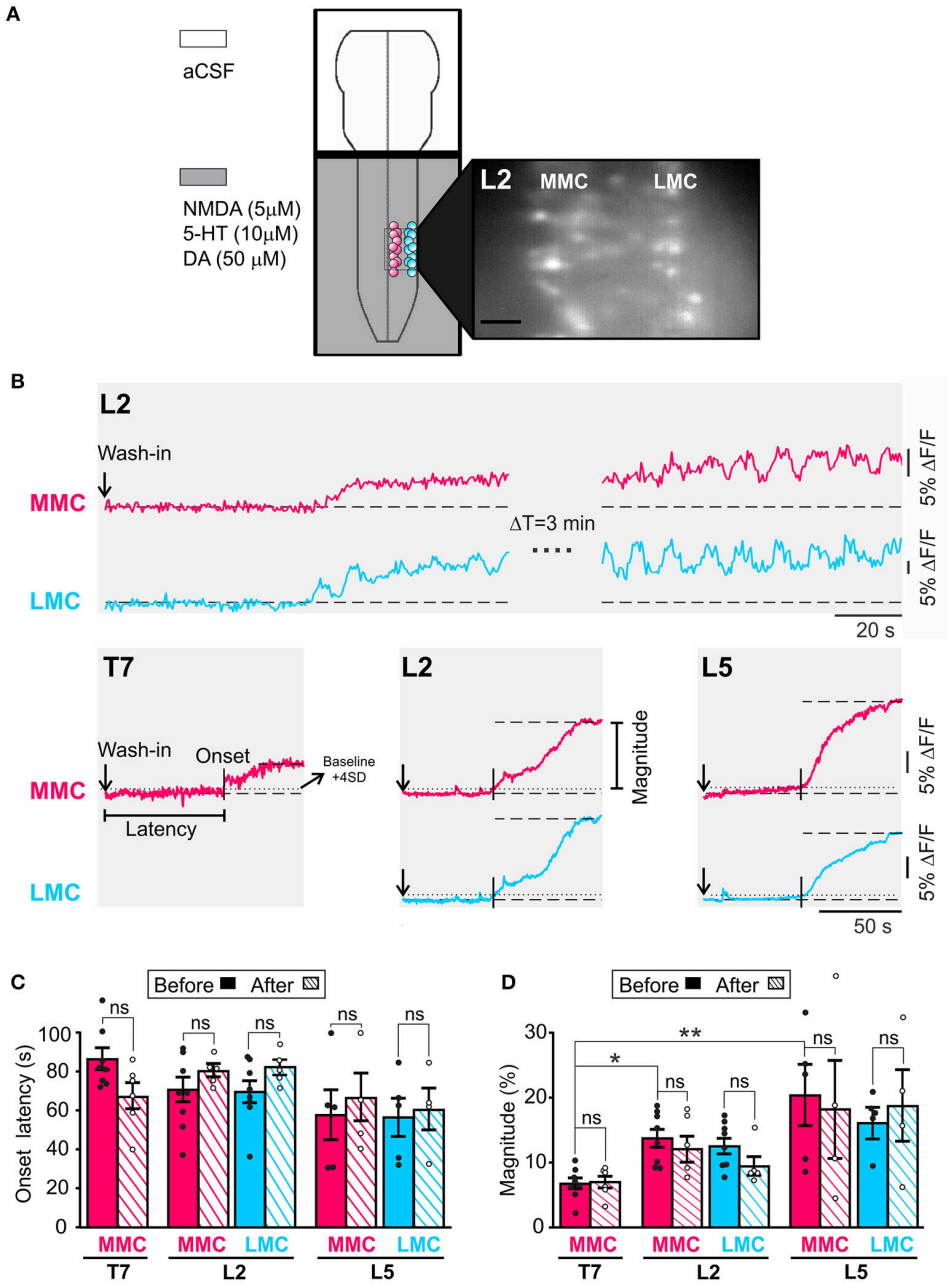
An initial period of tonic activity accompanies rhythmic activity both in MMC and in LMC MNs. **(A)** Schematic representation of the experimental arrangement. The recording chamber was partitioned in two compartments with a plastic bridge sealed with petroleum jelly. The brain stem compartment (top) was superfused with aCSF while the spinal cord compartment (bottom) was superfused with aCSF plus NMDA (5 μM), 5HT (10 μM), and DA (50 μM). The presence of the neurochemicals is indicated by the gray shading. MNs in the MMC (red circles) and LMC (blue circles) were loaded with fluorescent calcium indicator CaGDA (see Methods). Inset: Photomicrograph of CaGDA loaded MMC and LMC MNs in L2. Scale bar is 25 μm. **(B)** Top: Changes in fluorescence following the arrival of the neurochemicals to the spinal cord compartment in a single MMC MN (red waveform) and LMC MN (blue waveform). The recording bouts on the left show the slow building up of the tonic component of LLA in each MNs. The recording bouts on the right, which were acquired 3 min later, show the same tonic component as it reaches maximal magnitude (plateau). Bottom: Population responses (large ROI covering the entirety of the motor columns) showing the tonic component of LLA in MMC MNs of T7, L2, and L5 segments and LMC MNs of L2 and L5 segment. Vertical arrows: Time of application of neurochemicals. Vertical bars: Onset latency. Horizontal dashed line: Mean baseline fluorescence. Horizontal dotted line: Mean baseline fluorescence +4 SD. **(C)** Bar graph showing the mean onset latency of the tonic component in T7, L2, and L5 MNs before (solid bars) and after (hatched bars) removal of the brain stem. Each bar is a grand average across all experiments (individual points). Mean onset latencies of tonic activity were similar before and after removal of the brain stem (Wilcoxon matched-pairs test, T7 MMC^before^ vs. MMC^after^ *p* = 0.22; L2 MMC^before^ vs. MMC^after^ *p* = 0.31; L5 MMC^before^ vs. MMC^after^ *p* = 0.25; L2 LMC^before^ vs. LMC^after^ *p* = 0.31; L5 LMC^before^ vs. LMC^after^ *p* > 0.99). **(D)** Bar graph showing the magnitude of the tonic component in T7, L2, and L5 MNs before (solid bars) and after (hatched bars) removal of the brain stem. Each bar is a grand average across all experiments (individual points). The mean magnitudes of tonic component were similar before and after removal of the brain stem (Wilcoxon matched-pairs test, T7 MMC^before^ vs. MMC^after^ *p* = 0.84; L2 MMC^before^ vs. MMC^after^ *p* = 0.88; L5 MMC^before^ vs. MMC^after^ *p* > 0.99; L2 LMC^before^ vs. LMC^after^ *p* > 0.99; L5 LMC^before^ vs. LMC^after^ *p* > 0.99). ^*^p < 0.05, ^**^p < 0.01.

An initial, slowly rising, tonic plateau of activity often preceded rhythmic LLA. Its onset was detected using a threshold function (Clampfit 10.4, Molecular Devices, USA) set to average baseline fluorescence + 4SD (horizontal dotted lines in Figure 1B) whereas its magnitude was quantified as the average plateau fluorescence (measured once the plateau had stabilized) minus the average baseline fluorescence. The baseline fluorescence and the plateau fluorescence were average over a 5 s-period.

##### Rhythmic component

Although cyclic changes in fluorescence were often observed within 3 min of neurochemicals application, we only analyzed recordings obtained ≥20 min (stable rhythm).

*Frequency of occurrence of double-peak bursts in MMC MNs*. Double-peak bursts were defined as bursts with a transient dip in fluorescence (>30% decrease from the highest peak value and duration of at least 1 s) about midway through their crest (**Figure 3A**). The proportion of double-peak bursts was assessed by compiling their numbers during 2 min- recording sessions (2–5 MMC MNs per experiment).

*Rhythm frequency and burst durations*. The onset and offset of the locomotor bursts in individual MNs were detected using a threshold function (single channel search function in Clampfit 10.4) set at 30% of the peak value (Tazerart et al., 2007). Times between consecutive onsets were used to determine the duration of each locomotor cycle and assess rhythm frequency whereas times between onsets and offsets were used to calculate burst durations.

*Temporal relationships and coupling strengths*. Temporal relationships and coupling strengths between pairs of MNs were determined for MNs of the same motor column (intra-columnar) and MNs of different motor columns (inter-columnar). For these analyses, we used “Spinalcore,” a program for signal processing and analyses in frequency/time domain of stationary and non-stationary time series (Mor and Lev-Tov, 2007). Briefly, the calcium signal from each individual MN was decomposed in the frequency domain over the time course of the recordings using a continuous Morlet wavelet transform (10 octaves per scale). Then, using coherent cross-power wavelet transform (CXWT, spectrograms in **Figure 6A**), we determined if two individual signals had 1) common bands of high-power frequencies and 2) a phase relationship that was consistent over time. The degree of consistency of the phase relationship (coupling strength or coherence) ranged from zero to one for highly coherent signals. The high-power frequencies band of interest in the CXWT was selected (white rectangle in spectrograms of **Figure 6A**) and used to extract rhythm frequency, phase differences and coupling strengths (coherence) between pairs of MNs. Rhythm frequencies and coupling strengths were presented as bar graphs and phase differences as circular plots.

### Statistics

Data are reported as mean±SEM, unless indicated otherwise. Tests of significance were performed on the grand averages across preparations and included: the paired *t*-test, unpaired *t*-test and one-way ANOVA followed by Holm-Sidak *post-hoc* multiple comparison. When the assumption of normality was violated, we instead used the Fisher's exact-test, Wilcoxon matched-paired test, Mann-Whitney test and Kruskal-Wallis test followed by Dunn's multiple comparison. These tests were performed using GraphPad Prism 6 (San Diego, CA, USA). To test mean phase differences, we performed Watson–Williams *F*-test using Oriana (Kovach Computing Services, Pentraeth, UK). Significance level was set at *p* < 0.05.

## Results

We analyzed optical recordings from more than 4000 individual MNs in the T7, L2, and L5 segments during drug-induced LLA. To achieve single-cell resolution each segment was investigated in different preparations. Below, sample sizes “*n*” refer to the number of animals.

### Initial tonic activity

An initial period of tonic activity preceding rhythmic activity has been reported during drug-induced LLA in the isolated spinal cord of the neonatal rat (Kjaerulff and Kiehn, 1996; Beliez et al., 2015) and during MLR-evoked fictive locomotion in adult the decerebrate cat and rat (Perreault et al., 1999; MacDonell et al., 2015). However, this tonic activity has not been described in the mouse. Here, we measured tonic activity in individual MNs in T7, L2, and L5 segments (5–10 MNs per motor column) and show that tonic activity develops both in MMC and in LMC MNs (Figure 1, *n* = 21). In both MN groups, tonic activity appeared within less than 2 min and increased until it reached a plateau (Figure 1B). Few minutes after that, rhythmic activity started. Occasionally, tonic and rhythmic activity would appear concomitantly (LMC trace in Figure 1B). On average, the magnitude of the tonic activity in MMC MNs was significantly smaller in T7 than that in L2 and L5 (Figure 1D, Kruskal-Wallis test followed by Dunn's multiple comparison, T7 vs. L2, *p* = 0.02; T7 vs. L5, *p* = 0.01), suggesting an inter-segmental difference between thoracic and lumbar segments. However, in the absence of dependable measure for difference in excitability between preparations, this suggestion must remain tentative.

Pairwise comparisons before and after removal of the brain stem (*n* = 15) revealed no significant differences in mean onset latency or magnitude (compare solid and hatched bars in Figures 1C,D, see figure legend for *p*-values). Since the presence of the brain stem did not appear to change the inherent capability of the spinal cord to generate tonic activity, we suggest that initial tonic activity during drug-induced LLA is organized largely by neural networks in the spinal cord.

### Rhythmic locomotor-like activity

Rhythmic activity was analyzed in T7, L2, and L5 segments (*n* = 10 animals per segment) before and after removal of the brain stem. Measurements were performed on individual MNs (10 MNs per motor column) and included rhythm frequency, burst durations, phase relationships and strengths of coupling between MNs. Data presented below include the first measurements of rhythmic locomotor activity in MMC MNs of the brain stem-spinal cord of the neonatal mouse.

#### Rhythm frequency and burst duration

**Figure 2:**
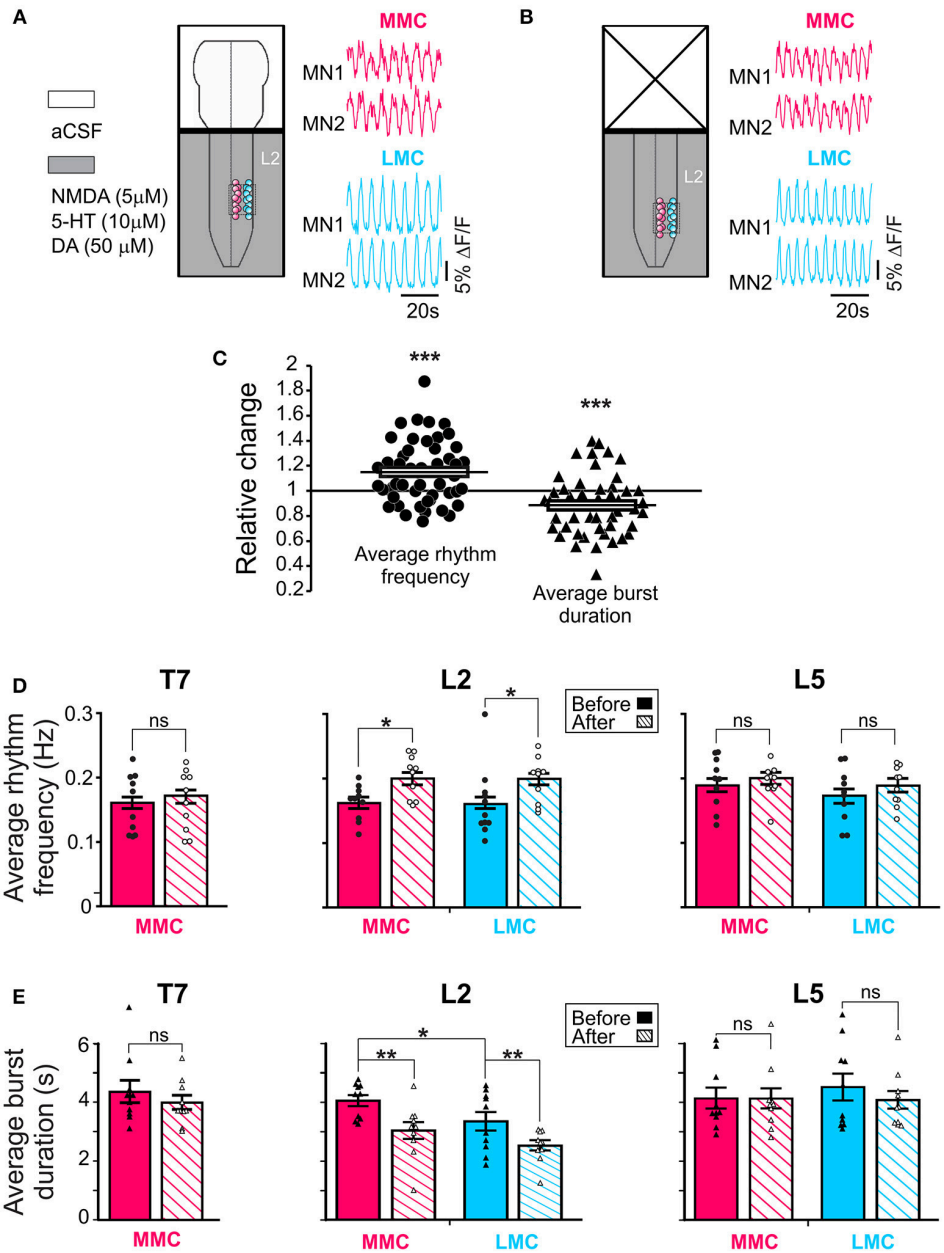
Regulation of the locomotor rhythm frequency and burst durations by the brain stem. Schematic representations of the experimental arrangement before **(A)** and after **(B)** removal of the brain stem shown together with corresponding set of waveforms displaying changes in fluorescence in two L2 MMC MNs (red) and two L2 LMC (blue) MNs (for other details, see Figure 1A). The same four MNs are shown in the two panels. **(C)** Pooled data from 50 motor columns (10 motor columns in T7, 20 motor columns in L2 and in L5) showing relative change in average rhythm frequency (solid circles) and burst duration (solid triangles) after removal of the brain stem. **(D,E)** Bar graphs showing the average rhythm frequency and average burst duration in MMC (red) and LMC MNs (blue) in the individual segments before and after removal of the brain stem (solid and hatched bars, respectively). Each bar is a grand average across all experiments. The increase in rhythm frequency after removal of the brainstem was statistically significant in L2 (paired *t*-test, L2 MMC^before^ vs. MMC^after^ *p* = 0.003; L2 LMC^before^ vs. LMC^after^ *p* = 0.048; T7 MMC^before^ vs. MMC^after^ *p* = 0.79; L5 MMC^before^ vs. MMC^after^ *p* = 0.86; L5 LMC^before^ vs. LMC^after^ *p* = 0.26). Removal of the brain stem also significantly reduced the average burst durations in L2 (paired *t*-test, L2 MMC^before^ vs. MMC^after^ *p* = 0.003; L2 LMC^before^ vs. LMC^after^ *p* = 0.004; T7 MMC^before^ vs. MMC^after^ *p* = 0.26; L5 MMC^before^ vs. MMC^after^ *p* = 0.97; L5 LMC^before^ vs. LMC^after^ *p* = 0.34). ^*^p < 0.05, ^**^p < 0.01, ^***^p < 0.001.

The rhythm frequencies and burst durations were assessed in all three segments and, when eligible, in both MMC and LMC MNs (Figure 2). Analyses were performed on both combined (Figure 2C) and individual segments/columns (Figures 2D,E).

Before removal of the brain stem, the average frequencies ranged between 0.11 and 0.31 Hz without significant difference in average frequencies between the segments (one-way ANOVA test followed by a Holm-Sidak multiple comparison, all *p* > 0.17). Removal of the brain stem increased the average rhythm frequency (solid circles in Figure 2C, paired *t*-test, *p* = 0.0008). When motor columns were analyzed separately, L2 showed that the increase in frequency occurred both in MMC and LMC (Figure 2D, solid vs. hatched bars, see figure legend for *p*-values).

We then tested whether the increase in locomotor frequency might have arisen from a reduction in locomotor burst durations. Prior to removal of the brain stem, the average burst durations ranged between 3.3 and 4.5 s without significant difference in average burst duration between segments (One-way ANOVA test followed by a Holm-Sidak multiple comparison, all *p* > 0.85). When motor columns were analyzed separately, L2 showed significantly longer burst durations in MMC than LMC MNs (paired *t*-test, *p* = 0.04). Removal of the brain stem reduced the average burst duration (solid triangles in Figure 2C, paired *t*-test, *p* = 0.0007). Again, when motor columns were analyzed separately, L2 showed that the reduction in burst duration occurred both in MMC and LMC (Figure 2E, solid vs. hatched bars, see figure l egend for *p*-values). Thus, it is likely that the increase in rhythm frequency after removal of the brainstem arose from a reduction in locomotor burst durations in both MMC and LMC MNs.

**Figure 3:**
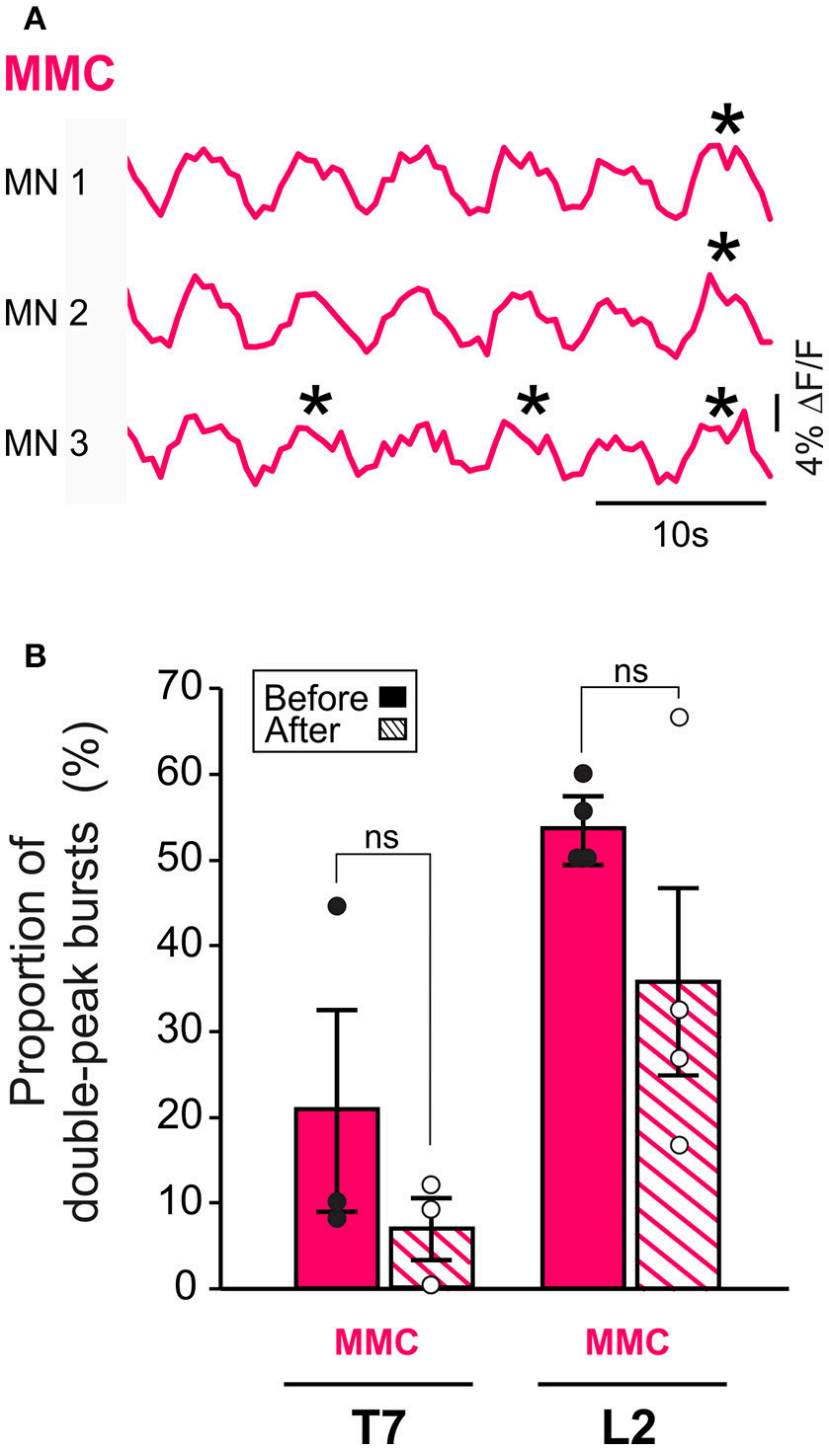
MMC MNs display double-peak bursts during LLA. **(A)** Changes in fluorescence in three individual MNs in the left MMC (top) and right MMC (bottom). *Asterisks* indicate double-peak bursts (see Methods). **(B)** Bar graphs showing the mean frequency of occurrence of double-peak bursts in MMC MNs of T7 and L2 before (solid bars) and after (hatched bars) removal of the brain stem. Each bar is a grand average across all experiments (individual points).

Additionally, removal of the brain stem eliminated the difference in L2 burst duration between MMC and LMC MNs seen in the brain stem-attached preparation (paired *t*-test, *p* = 0.10). This observation prompted us to look more closely at the shape of the bursts in MMC MNs. We observed that some of the bursts in MMC MNs displayed double-peak, similar to those reported previously in the L3–L5 segments of the isolated spinal cord (O'Donovan et al., 2008). Therefore, we wanted to test whether regulation of double-peak bursts by the brain stem could contribute to the reduction in burst duration in L2 MMC MNs. We compared the proportion of double-peak bursts during 2 min-recording sessions before and after removal of the brain stem (*n* = 7, Figure 3). Comparisons were made for L2 and T7 segments. Before removal of the brain stem, we observed double-peak bursts in MMC MNs (asterisks in Figure 3A, see also MMC traces in Figure 2) in both L2 and T7, albeit at a lower proportion in T7 (54% in L2 vs. 21% in T7, Figure 3B, solid bars). After removal of the brain stem (Figure 3B, hatched bars), the proportion of double-peak bursts in L2 decreased to 36%. However, the decrease was not statistically significant (Fisher's exact test, *p* = 0.07) nor was it specific to L2 (decrease to 7% in T7, Fisher's exact test, *p* = 0.11).

Altogether, these findings suggest that the brain stem contributes to the regulation of the rhythm frequency and burst duration of both MMC and LMC MNs during drug-induced LLA. When columns were analyzed separately, the threshold for significance was reached in L2 but not in T7 or L5. This is likely because MNs in this segment display tighter temporal relationships (see Supplemental Figure 1 and section below). We also observed a reduced incidence of double-peak bursts in L2 MMC MNs after removal of the brain stem but this does not appear to contribute to the reduction in locomotor burst durations in these MNs.

#### Phase relationships and coupling strengths between MNs of the same motor column

**Figure 4:**
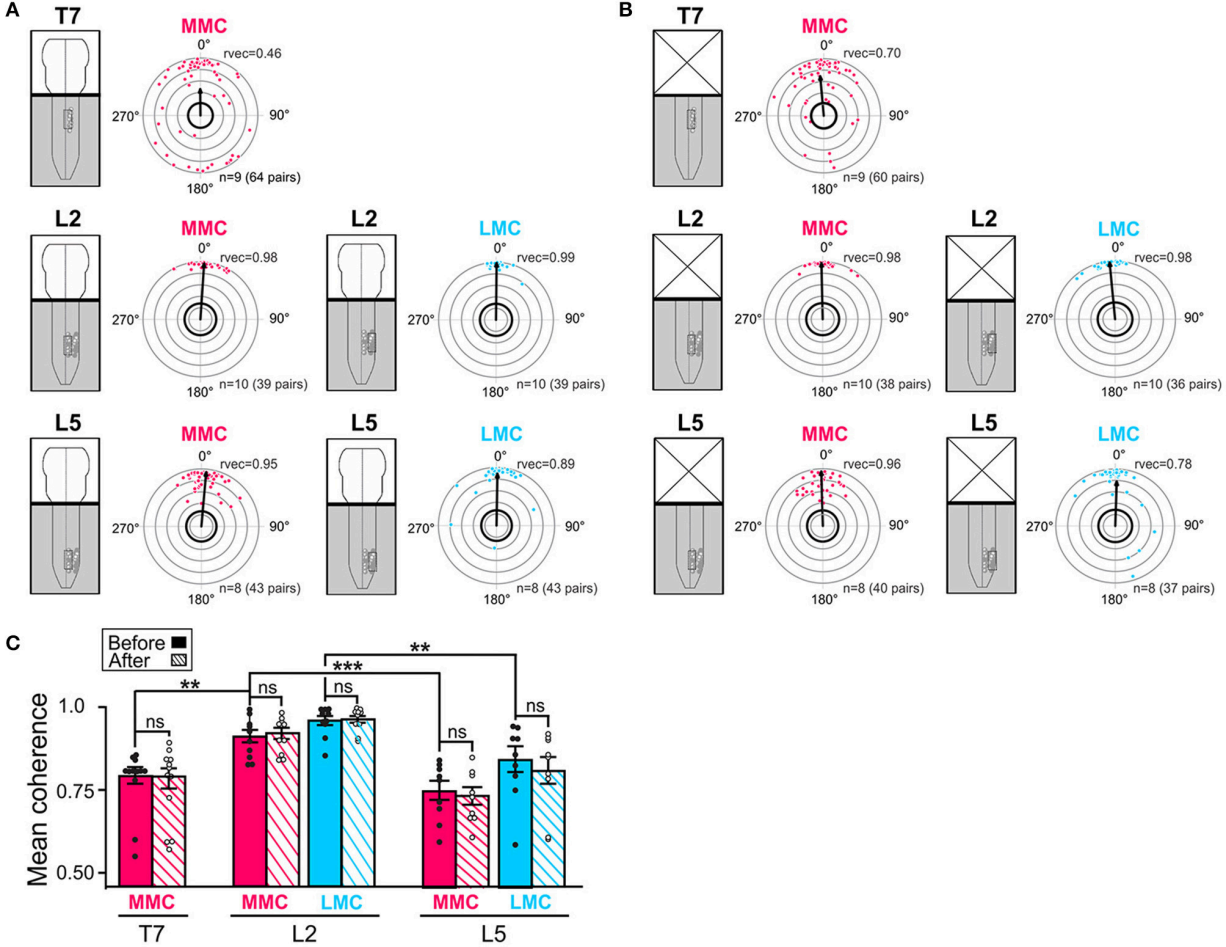
Temporal relationships and coupling strengths (coherence) between MNs of the same motor column before and after removal of the brain stem. **(A,B)** Intra-columnar phase relationships between pairs of MNs in T7 MMC (top), L2 MMC and LMC (middle) and L5 MMC and LMC (bottom) before and after removal of the brain stem, respectively. Phase relationships between MN pairs are shown as phase differences in circular plots. Individual dots represent individual MN pairs (red = >MMC, blue = >LMC). The direction of the vectors (arrows) indicates mean phase difference (0° = >synchronous, 180° difference = >alternating) whereas the length of the vectors (rvec) indicates the extent to which the phase differences are clustered around the mean on a scale from 0.0 (origin) to 1.0 (outermost gray circle). The circle with thick black outline indicates the *p* < 0.05 level of statistical significance as determined by the Rayleigh test. Number of experiments and number MN pairs analyzed indicated at the bottom of each circular plots. The lower average vector length “rvec” in T7 indicates a more widespread distribution of phase differences between MNs in this segment than in L2 or L5. Phase differences were clustered in two groups around 0 and 180°. The changes in mean phase vectors after removal of the brainstem were not statistically significantly (Watson–Williams *F*-test, T7 MMC^before^ vs. MMC^after^ *F* = 0.83, *p* = 0.38; L2 MMC^before^ vs. MMC^after^ *F* = 2.25, *p* = 0.15; L2 LMC^before^ vs. LMC^after^ *F* = 2.53, *p* = 0.13; L5 MMC^before^ vs. MMC^after^ *F* = 1.28, *p* = 0.28; L5 LMC^before^ vs. LMC^after^ *F* = 1.28, *p* = 0.63). **(C)** Bar graph showing the mean coherence before (solid bars) and after (hatched bars) removal of the brain stem. The coupling between MNs was significantly tighter in L2 than in T7 or L5 both before and after removal of the brain stem (Kruskal-Wallis test followed by a Dunn's multiple comparison, L2 MMC^before^ vs. T7 MMC^before^ *p* = 0.007; L2 MMC^before^ vs. L5 MMC^before^ *p* = 0.0004; L2 LMC^before^ vs. L5 LMC^before^ *p* = 0.001; L5 MMC^before^ vs. T7 MMC^before^ *p* > 0.99; Wilcoxon matched paired test, T7 MMC^before^ vs. MMC^after^ *p* = 0.82; L2 MMC^before^ vs. MMC^after^ *p* = 0.56; L2 LMC^before^ vs. LMC^after^ *p*> 0.99; L5 MMC^before^ vs. MMC^after^ *p* = 0.73; L5 LMC^before^ vs. LMC^after^ *p* = 0.57). ^**^p < 0.01, ^***^p < 0.001.

We then sought to determine the temporal relationships and strengths of coupling between MNs within individual motor columns. Several pair of MNs were analyzed within each motor column. Each pair consisted of a reference MN and another, randomly selected, MN within the motor column. The reference MN was selected based on its rostral most location in the field of view. All intra-columnar phase relationships (mean phase vector) and coupling strengths (mean coherence) analyzed in T7, L2, and L5 are shown in Figure 4.

Before removal of the brain stem (Figure 4A), all the mean phase vectors were close to 0°, suggesting that MN pairs discharged synchronously. However, the mean vector length “rvec” was lower in T7 MMC compared to L2 or L5 MMC, indicating a more widespread distribution of phase differences. As shown in the circular plot of T7, this widespread distribution was not the result of a uniform distribution of phase differences around the circle (Watson's *U*2-test, *p* < 0.01), but rather a clustering of phase differences into two groups separated by 180°; one large cluster of MN pairs discharging synchronously and a smaller cluster of MN pairs discharging out-of-phase. Removal of the brain stem did not significantly alter the mean phase vectors in any of the segment (Figure 4B, see figure legend for *p*-values).

The mean coherences between MN pairs ranged between 0.75 and 0.96 and were significantly higher between MNs of L2 motor columns than between MNs of the T7 and L5 motor columns (Figure 4C, filled bars, see figure legend for *p*-values). The lower coherence between T7 MMC MNs T7 may be attributable to the presence of less well-correlated MN pairs (dots close to the thick, black outline circle in circular plots). This is consistent with the recent finding of Beliez et al. (2015) who reported a substantial number of non-locomotor-driven MNs in this segment. Removal of the brain stem did not significantly alter the mean coherences (Figure 4C, hatched bars; see figure legend for *p*-values).

Altogether, these data indicate that MNs within L2 and L5 motor columns discharge in phase with each other during drug-induced LLA in the brain stem-spinal cord preparation. In T7 motor columns, many MNs discharge in phase while a substantial number discharge out-of-phase. We also found that the temporal relationships between MNs were tightest in L2, a finding that is compatible with a higher potential for rhythmogenesis (see Discussion). Removal of the brain stem, despite its clear effect on the locomotor frequency and burst durations, did not significantly affect intra-columnar phase relationships or coupling strengths between MNs.

#### Phase relationships and coupling strengths between MNs of different motor columns

**Figure 5:**
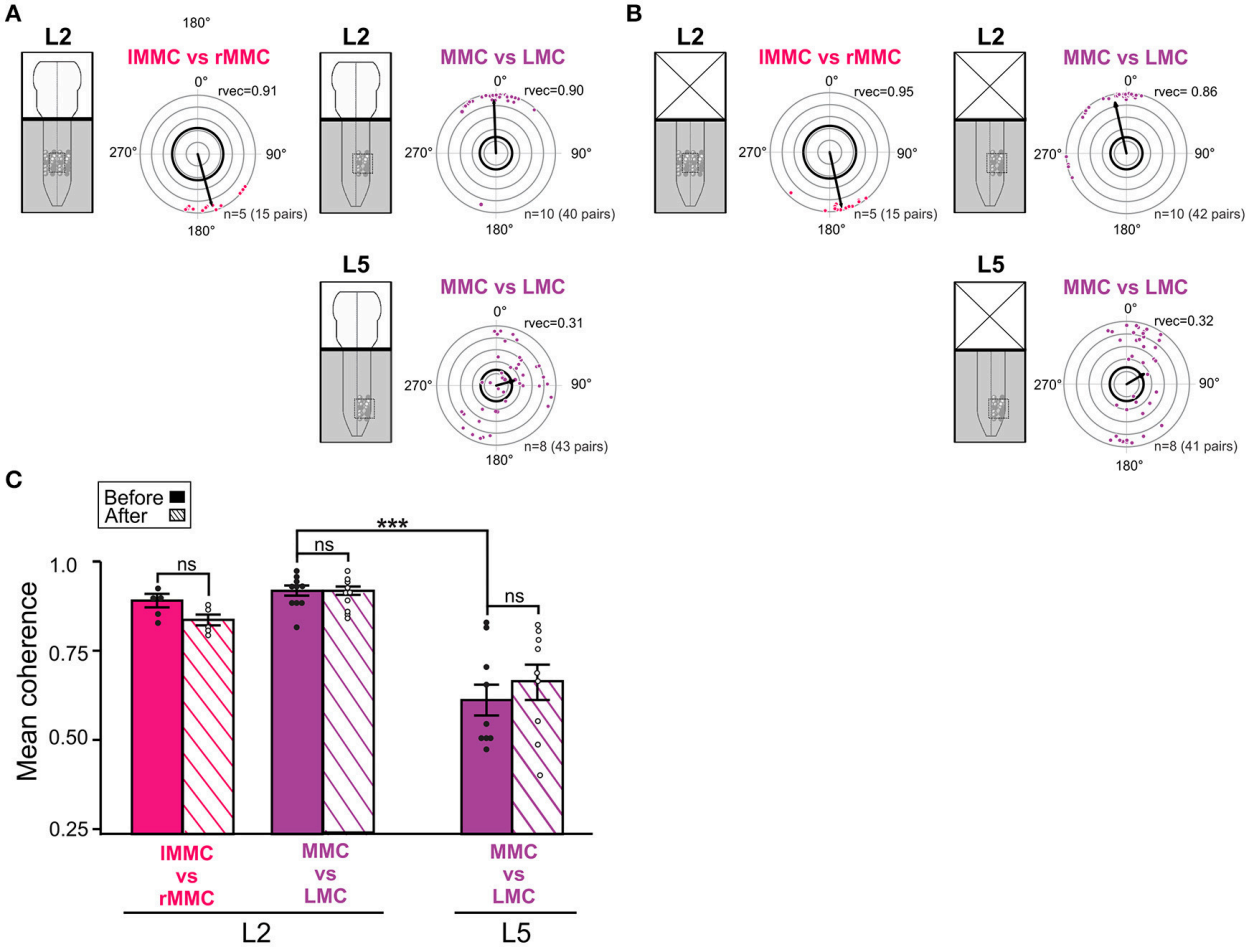
Temporal relationships and coupling strengths between MNs of different motor columns before and after removal of the brain stem. **(A,B)** Inter-columnar phase relationships between pairs of MNs in left and right MMC in L2, MMC and LMC in L2, and MMC and LMC in L5 before and after removal of the brain stem. Phase relationships between MMCs are shown in red and phase relationships between MMC and LMCs are shown in purple. Removal of the brain stem did not significantly change the mean phase differences between MNs (Watson–Williams *F*-test, L2 lMMC/rMMC^before^ vs. lMMC/rMMC^after^ *F* = 0.03, *p* = 0.87; L2 MMC/LMC^before^ vs. MMC/LMC^after^ *F* = 0.60, *p* = 0.45; L5 MMC/LMC^before^ vs. MMC/LMC^after^ *F* = 0.21, *p* = 0.65). **(C)** Bar graph showing mean coherence before (solid bars) and after (hatched bars) removal of the brain stem. The strengths of the coupling between MNs was not significantly affected by removal of the brain stem (Wilcoxon matched-pairs test, L2 lMMC/rMMC^before^ vs. lMMC/rMMC^after^ *p* = 0.19; Kruskal-Wallis test followed by a Dunn's multiple comparison, L2 MMC/LMC^before^ vs. L2 MMC/LMC^after^ *p* > 0.99; L5 MMC/LMC^before^ vs. L5 MMC/LMC^after^ *p* > 0.99). ^***^p < 0.001. For other details, see Figure 4.

We next examined the temporal relationships and strengths of coupling between MNs in different motor columns. Inter-columnar relationships were determined between MNs of the left and right MMCs in L2 and between MNs in the MMC and LMC in both L2 and L5. All inter-columnar phase relationships (mean phase vector) and coupling strengths (mean coherence) are shown in Figure 5.

Before removal of the brain stem (Figure 5A), the mean phase vector between left and right L2 MMC MNs was around 180°, indicating left/right alternation between axial MNs. In contrast, the mean phase vector between MMC and LMC MNs on the same side of L2 was close to 0°, indicating synchronous activation. In L5, the mean phase vector between MMC and LMC MNs was close to 90°, differing significantly from the mean phase vector in L2 (Watson–Williams *F*-test, *F* = 9.462, *p* = 0.007). The mean vector length in L5 was also smaller than in L2, indicating a more widespread distribution of phase differences. However, the distribution was not uniform (Watson's *U*2-test, *p* < 0.01) and close inspection of the L5 circular plots revealed a clustering of phase differences into at least two groups; one group where MMC and LMC MNs discharged mostly in-phase (quadrant 0–90°) and another one group where MMC and LMC MNs discharged out-of-phase (around 180°).

In addition, several MMC/LMC pairs were less, or not, correlated (dots close or within the thick black outline circle). Removal of the brain stem did not significantly change the mean phase vectors between left and right L2 MMC MNs or between MMC and LMC MNs in L2 and L5 (Figure 5B, see figure legend for *p*-values).

The coherence between MMC and LMC MNs was found to be significantly lower in L5 than in L2 (MMC vs. LMC solid bars in Figure 5C, Kruskal-Wallis test followed by a Dunn's multiple comparison, *p* = 0.0005), suggesting a much more labile coupling between MMC and LMC MNs in L5 than in L2. This might be attributable to the presence of some poorly correlated MNs in the MMC (see previous section) rather than in the LMC. Removal of the brain stem did not significantly change the coherence between MMC and LMC MNs either in L2 or L5 (Figure 5C, hatched bars see figure legend for *p*-values).

Altogether, these findings demonstrate left/right alternation between L2 MMC MNs, synchrony between L2 MMC and LMC MNs, and combinations of synchrony and alternation between L5 MMC and LMC MNs during drug-induced LLA in the brain stem-spinal cord preparation. As intra-columnar relationships, inter-columnar relationships were not significantly affect by removal of the brain stem, suggesting that during drug-induced LLA these relationships are organized largely by the spinal networks.

### Effect of reversible cooling of the brain stem on L2 rhythm frequency

To investigate whether activity in brain stem neurons contributed to the increase in L2 rhythm frequency observed after removal of the brain stem, we cooled down the brain stem to 18°C (*n* = 4). At this temperature, activity in neuronal somata is blocked and transmission along axons is greatly reduced (Brooks, 1983).

**Figure 6:**
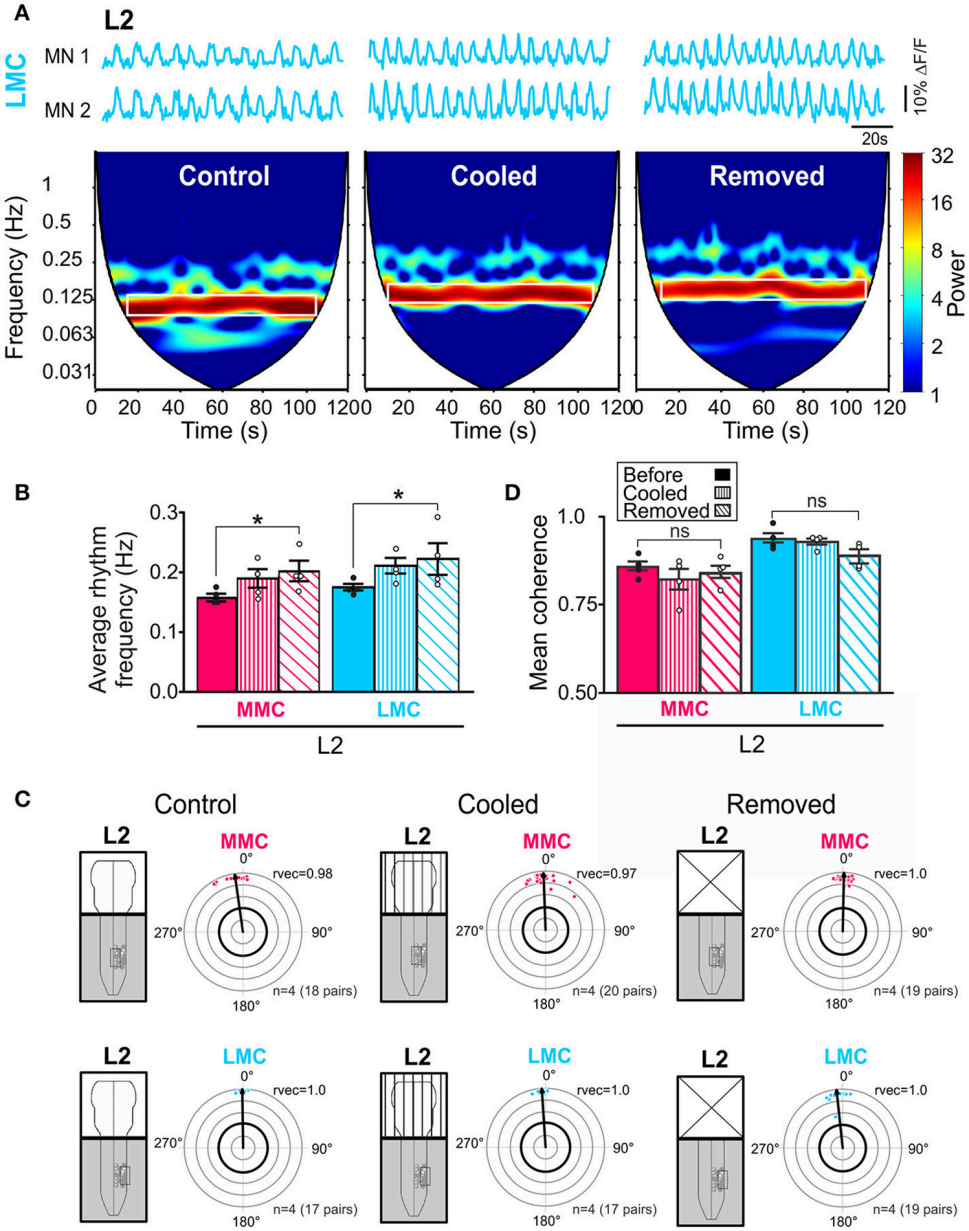
Reduced brain stem activity also increases locomotor rhythm frequency. **(A)** Three sets of waveforms showing changes in fluorescence in two L2 LMC MNs before and during cooling of the brain stem and after removal of the brain stem. Each set of waveforms is shown with corresponding coherent cross-wavelet transform (CXWT) spectrogram (see Methods). The band of high-power (red) frequencies is marked with a white rectangle indicates a significantly coherence between the two signals in the neighborhood of the locomotor rhythm frequency. The two bottom white regions on either side of the V-shaped “cone of influence” indicate spectra regions not analyzed due to edge effects (Mor and Lev-Tov, 2007). **(B)** Bar graphs showing the average rhythm frequency in each motor column before (solid bars) and during cooling (vertically hatched bars) of the brain stem, and after removal of the brain stem (obliquely hatched bars). The increase after cooling was not as large as the increase after complete removal of the brain stem but it was observed in all experiments in both MMC and LMC MNs (Friedman test followed by Dunn's multiple *post-hoc* comparison, MMC^control^ vs. MMC^cooled^ *p* = 0.23 MMC^control^ vs. MMC^removed^ *p* = 0.04; MMC^cooled^ vs. MMC^removed^ *p* > 0.99; LMC^control^ vs. LMC^cooled^ *p* = 0.23; LMC^control^ vs. LMC^removed^ *p* = 0.04; LMC^cooled^ vs. LMC^removed^ *p* > 0.99). **(C)** Circular plots showing the effect of cooling and removing the brain stem on the intra-columnar phase differences (Watson–Williams *F*-test, MMC^control^ vs. MMC^cooled^ *F* = 0.69, *p* = 0.46; MMC^control^ vs. MMC^removed^ *F* = 2.23, *p* = 0.19; MMC^cooled^ vs. MMC^removed^ *F* = 0.18, *p* = 0.68; LMC^control^ vs. LMC^cooled^ *F* = 2.28, *p* = 0.18; LMC^control^ vs. LMC^removed^ *F* = 5.56, *p* = 0.06 LMC^cooled^ vs. LMC^removed^ *F* = 1.07, *p* = 0.34). **(D)** Bar graphs showing the effect of cooling and removal the brain stem on the intra-columnar coupling strengths (Friedman test followed by a Dunn's multiple comparison, MMC^control^ vs. MMC^cooled^ *p* = 0.47; MMC^control^ vs. MMC^removed^ *p*> 0.99; MMC^cooled^ vs. MMC^removed^ *p*> 0.99; LMC^control^ vs. LMC^cooled^ *p*> 0.99; LMC^control^ vs. LMC^removed^ *p* = 0.23; LMC^cooled^ vs. LMC^removed^ *p* = 0.47). ^*^p < 0.05.

Cooling of the brain stem increased the locomotor rhythm frequency (Figure 6A), an effect that was reversible. In the spectrograms of Figure 6A, the increase is shown as a shift of the high-power frequencies band (white rectangle) toward higher frequencies. The increase was not as large as the increase observed after complete removal of the brain stem but it was observed in all experiments both in MMC and LMC MNs (Figure 6B, see figure legend for *p*-values). We also analyzed intra-columnar phase relationships and strengths of coupling during cooling and, as shown in Figures 6C,D, the effects of cooling on these parameters were indistinguishable from the effects observed after removal of the brain stem (see figure legend for *p*-values).

These results demonstrate a similar effect of cooling and removal of the brain stem on the locomotor rhythm and support a contribution from brain stem descending neurons.

## Discussion

### General findings

In this study, we characterized for the first time the activities of trunk (MMC) and hindlimb (LMC) MNs during drug-induced fictive locomotion in the brain stem-spinal cord preparation of the neonatal mouse. We found that (1) trunk and hindlimb MNs display a preparatory period of tonic activity prior to rhythmic activity, (2) trunk MNs in T7 burst either in-phase or out-of-phase during rhythmic activity whereas trunk MNs in L2 or L5 burst in-phase, and (3) most trunk MNs in L2 burst in-phase with hindlimb MNs whereas in L5, several trunk MNs also burst out-of-phase with hindlimb MNs. Removal of the brain stem significantly increased the locomotor rhythm frequency with concomitant shortening in locomotor bursts. When individual segments were considered separately, the effects of removing the brain stem were significant in L2 but not T7 or L5. One interpretation of this result is that, in neonatal mouse, the brain stem targets its influence specifically on the thoracolumbar segments that have the highest potential for generating rhythmic locomotor activity (Ho and O'Donovan, 1993; Cazalets et al., 1995; Kjaerulff and Kiehn, 1996; Cowley et al., 2009; Hägglund et al., 2013).

Our work adds to a previous study in neonatal decerebrate mice in which an increase in locomotor rhythm frequency after removal of the brainstem was observed in L2 MNs (Gordon and Whelan, 2008). Both the recording method and the method used to induce fictive locomotion in this study differed from the methods used in the present study, suggesting that the increase in L2 locomotor rhythm frequency after removal of the brainstem is a robust phenomenon. In the adult decerebrate mice, spinalization also produces an increase in locomotor frequency (Meehan et al., 2012).

### Candidate subcortical descending systems

The increase in locomotor rhythm frequency after brainstem removal may be attributable to the loss of inputs from one or more brainstem descending systems. Prime candidates are the reticulospinal and the vestibulospinal systems (Grillner, 1981; Armstrong, 1988; Jordan, 1991; Zaporozhets et al., 2004; Jordan et al., 2008; Hägglund et al., 2010; Bretzner and Brownstone, 2013; Perreault and Glover, 2013).

Both the reticulospinal system and the lateral vestibulospinal system have the functional connectivity that would allow control over MMC and LMC MNs in the newborn mouse (Szokol et al., 2008; Szokol and Perreault, 2009; Kasumacic et al., 2010; Sivertsen et al., 2014). Although there is currently no available data on the discharge pattern of the reticulo- or vestibulospinal neurons during locomotor activity in the decerebrated mouse, earlier studies in adult decerebrate lampreys, guinea pigs, and cats indicate that the majority of these neurons discharge rhythmically during fictive locomotion (Kasicki et al., 1989; Bussières and Dubuc, 1992; Marlinsky, 1992; Perreault et al., 1993). Electrical or optogenetic stimulation to increase the ongoing locomotor discharge of reticulo- or vestibulospinal neurons in decerebrated preparations can lead to a prolongation of the locomotor cycle (Russell and Zajac, 1979; Vinay and Grillner, 1993; Perreault et al., 1994; Leblond and Gossard, 1997; Bouvier et al., 2015). Hence, loss of reticulo- and/or vestibulo-spinal inputs after spinal cord transection would be consistent with a decrease in locomotor cycle.

Monoaminergic descending systems such as the coerulospinal and raphespinal systems could also participate. The discharge pattern of monoaminergic descending neurons during locomotor activity has not been investigated in decerebrate neonatal mouse but available evidence from experiments in adult cats suggests that a majority of raphespinal neurons are tonically active during locomotion (Veasey et al., 1995; Noga et al., 2017). Serotonin prolongs locomotor cycle and axial MN burst durations in adult decerebrate lampreys (Buchanan, 2011). Conversely, serotoninergic receptor antagonists reduce the locomotor burst and cycle duration in limb MNs in decerebrate rats (Cabaj et al., 2017). Thus, loss of tonic raphespinal input after spinal transection would also be consistent with a decrease in locomotor cycle. However, a more precise assessment of the relative contribution of the different supraspinal neuronal groups must await a future characterization of their locomotor discharge patterns in the mouse.

### Tonic activity, double-peak bursts in axial MNs and trunk-hindlimb coordination

We report tonic and rhythmic LLA in axial MNs of T7, L2, and L5 segment. The tonic activity in axial MNs have similar spatiotemporal characteristics as the tonic activity in hindlimb MNs, a finding that is consistent with the idea that tonic activity in MNs act as a constant background for rhythmic activity (Kjaerulff and Kiehn, 1996). Tonic activity was not affected by removal of the brain stem, suggesting that it is produced by neuronal networks located in the spinal cord. We speculate that these spinal networks may play an important role in production of the postural responses that accompany locomotor movement by setting the level of excitability of the spinal neurons that form the locomotor rhythm generator.

We report single locomotor burst and double-peak locomotor bursts in axial MNs. The functional significance of the double-peak bursts is unclear but *double*-*bursts* in trunk muscles in various adult mammalian species has been associated with a multifunctional role in mobilization and stabilization of the trunk and pelvis, a requirement for movement of the limbs during walking (Carlson et al., 1979; English, 1980; Thorstensson et al., 1982; Zomlefer et al., 1984; Ritter et al., 2001; Wada and Kanda, 2004; Wada et al., 2006; de Sèze et al., 2008; Ceccato et al., 2009; Schilling and Carrier, 2010). Double-peak locomotor bursts tend to be less common after removal of the brainstem. Interestingly, double-bursts in adult trunk muscles are also less frequent after spinal transection unless animals are provided with neurochemicals, training, or sensory afferent input activation (Koehler et al., 1984; Zomlefer et al., 1984; Barbeau and Rossignol, 1987). Future experiments on the development of double-peak bursts are required to determine if more parallels exist between double-peak bursts in neonates and double-bursts in adults, and ultimately inform about the possibility that double-peak bursts lead to the expression of full-fledge double-bursts in adults.

Our findings on the temporal dynamic of the locomotor activities between axial and hindlimb MNs add to previous single-cell resolution work in the isolated lumbar spinal cord and confirm a more complex coupling between axial and hindlimb MNs in the L5 (O'Donovan et al., 2008; Hinckley et al., 2015). Removal of the brain stem does not significantly affect inter-columnar phase relationships either in L2 or in L5, suggesting that trunk-hindlimb coordination in neonates greatly relies on propriospinal mechanisms. These mechanisms may include segmental as well as inter-segmental mechanisms (Falgairolle and Cazalets, 2007). Finally, in addition to their role in trunk-hindlimb coordination, thoracolumbar MNs innervating abdominal and pelvic musculature (Schrøder, 1980; Canon et al., 1991; Giraudin et al., 2008) may also assist respiratory exhalation during rhythmic motor activity (Iscoe, 1998; Hodges et al., 2007). Compatible with such a role, thoracolumbar MNs innervating abdominal muscles have recently been shown to display LLA during drug-induced fictive locomotion both before and after silencing of the brain stem (Le Gal et al., 2016).

## Conclusion

In the current study, we have assessed the influence of the brain stem on locomotor activity in axial and hindlimb spinal networks in newborn mammals. The study reveals an influence on the timing of the locomotor activity not only in hindlimb but also in axial motor pools. This influence on the axial and hindlimb spinal locomotor rhythm generating circuits may extend the range of frequencies over which these circuits operate. Having this ability early during development may be critical when learning how to walk is a major undertaking.

## Author contributions

Funding acquisition: M-CP; Conceptualization: M-CP and CJ-X; Supervision: M-CP; Methodology: CJ-X and M-CP; Experiments: CJ-X; Analyses: CJ-X and M-CP; Interpretation: M-CP and CJ-X; Writing of original draft: M-CP; Editing and approval of final version approval: M-CP and CJ-X.

### Conflict of interest statement

The authors declare that the research was conducted in the absence of any commercial or financial relationships that could be construed as a potential conflict of interest.
