# Supplementary material for: Influence of Brain Stem on Axial and Hindlimb Spinal Locomotor Rhythm Generating Circuits of the Neonatal Mouse
Source: Front Neurosci. 2018 Feb 9;12:53. doi: 10.3389/fnins.2018.00053 (PMC5811543; doi:10.3389/fnins.2018.00053)
Supplement: Supplementary file 1 [file DataSheet1.PDF]

Supplemental Figure 1

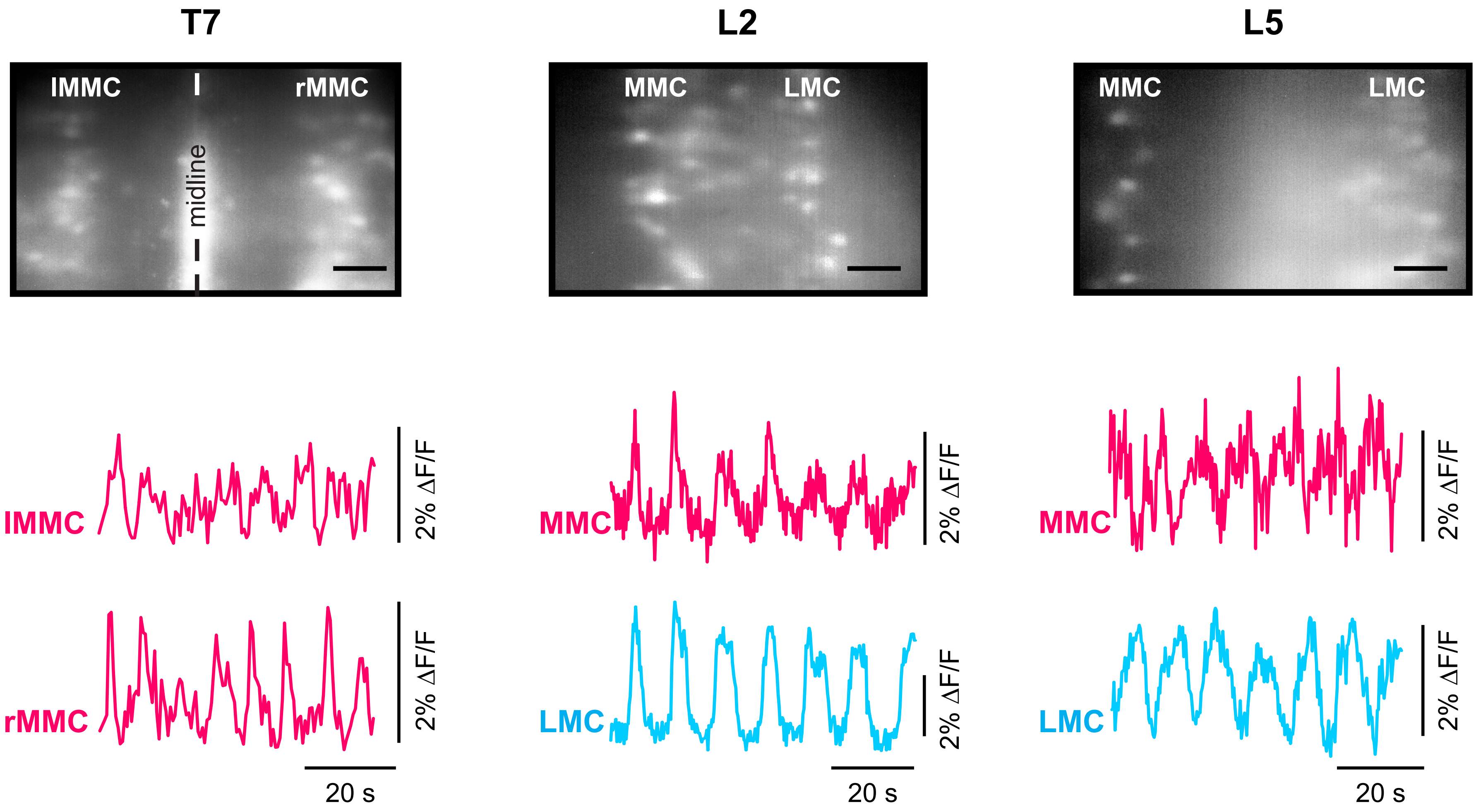

Supplemental figure 1. Rhythmic LLA in three different brain stem-spinal cord preparations. Top: Photomicrographs of CaGDA loaded MNs in T7, L2 and L5 segments. Scale bar is 25 µm. **Bottom**: Changes in fluorescence in left and right MMC in T7, MMC and LMC in L2 , and MMC and LMC in L5. For other details see Figure 1 in manuscript.
